# Novel Mechanism for an Old Drug: Phenazopyridine is a Kinase Inhibitor Affecting Autophagy and Cellular Differentiation

**DOI:** 10.3389/fphar.2021.664608

**Published:** 2021-08-04

**Authors:** Olivier Preynat-Seauve, Evelyne Bao-Vi Nguyen, Yvonne Westermaier, Margaux Héritier, Sébastien Tardy, Yves Cambet, Maxime Feyeux, Aurélie Caillon, Leonardo Scapozza, Karl-Heinz Krause

**Affiliations:** ^1^ Laboratory of Therapy and Stem Cells, Department of Diagnostics, Geneva University Hospitals, Geneva, Switzerland; ^2^ Department of Medicine, Faculty of Medicine, University of Geneva, Geneva, Switzerland; ^3^ Department of Pathology and Immunology, Faculty of Medicine, University of Geneva, Geneva, Switzerland; ^4^ Pharmaceutical Biochemistry Group, School of Pharmaceutical Sciences, Faculty of Science, University of Geneva, Geneva, Switzerland; ^5^ READS Unit, Faculty of Medicine, University of Geneva, Geneva, Switzerland

**Keywords:** phenazopyridine, kinase, phosphatidylinositol kinase, autophagy, differentiation

## Abstract

Phenazopyridine is a widely used drug against urinary tract pain. The compound has also been shown to enhance neural differentiation of pluripotent stem cells. However, its mechanism of action is not understood. Based on its chemical structure, we hypothesized that phenazopyridine could be a kinase inhibitor. Phenazopyridine was investigated in the following experimental systems: 1) activity of kinases in pluripotent stem cells; 2) binding to recombinant kinases, and 3) functional impact on pluripotent stem cells. Upon addition to pluripotent stem cells, phenazopyridine induced changes in kinase activities, particularly involving Mitogen-Activated Protein Kinases, Cyclin-Dependent Kinases, and AKT pathway kinases. To identify the primary targets of phenazopyridine, we screened its interactions with 401 human kinases. Dose-inhibition curves showed that three of these kinases interacted with phenazopyridine with sub-micromolar binding affinities: cyclin-G-associated kinase, and the two phosphatidylinositol kinases PI4KB and PIP4K2C, the latter being known for participating in pain induction. Docking revealed that phenazopyridine forms strong H-bonds with the hinge region of the ATP-binding pocket of these kinases. As previous studies suggested increased autophagy upon inhibition of the phosphatidyl-inositol/AKT pathway, we also investigated the impact of phenazopyridine on this pathway and found an upregulation. In conclusion, our study demonstrates for the first time that phenazopyridine is a kinase inhibitor, impacting notably phosphatidylinositol kinases involved in nociception.

## Introduction

Phenazopyridine [2,6-diamino-3-(phenylazo)pyridine] is used for its efficiency in the treatment of urinary urgencies and can be helpful in the context of urinary tract infection, interstitial cystitis, as well as medical procedures (endoscopy or surgery), which result in irritation and pain of the urinary tract. Phenazopyridine is generally well supported and sold as an over the counter medication in the United States. Side effects are generally benign except for rarely occurring methemoglobinemia (Murphy and Fernandez, 2018). No antibacterial effects are known for phenazopyridine, but analgesic effects on urinary pain without any known mechanism of action were observed. On a cellular level, the only published effects of phenazopyridine showed that the compound increases and synchronizes neural specification and maturation of Pluripotent Stem Cells (PSC) (Suter et al., 2009).

A hypothesis of mechanism of action comes from the chemical structure of phenazopyridine: the pyridine-2,6-diamine moiety of phenazopyridine is a typical kinase inhibition motif (Lin et al., 2005). To corroborate this hypothesis which was not supported by any prior study, we performed an inverse virtual screening on a diverse target set including several thousand proteins, including target classes of pharmaceutical interest. The high frequency of kinases in the high ranks of the interaction-score based ranking obtained from docking phenazopyridine into this large target panel confirmed the involvement of kinases.

*In silico* screening is a powerful approach for deciphering the interactions of small molecules with proteins. Classical structure-based virtual screening relies on *in silico* binding predictions of a wealth of compounds to a given protein target and aims at retrieving (specific) ligands. In contrast, inverse virtual screening is ligand-driven and aims at identifying possible targets for a given drug. We used this less known, reverse docking-based technique here to shed light on potential targets of phenazopyridine and establish binding mode hypotheses within three targets. Methodological considerations and successful examples of inverse virtual screening are discussed in detail in (Westermaier et al., 2015). *In silico* screening was for example successfully applied to kinases for tackling the broad promiscuity of inhibitors causing off-target side effects (Sterling and Williamson, 2008) and to the discovery of human secreted phospholipase A2 as the most likely targets for two inhibitors with µM affinities (Muller et al., 2006).

Kinases are prime drug targets and there is an increasing number of non-biased methods to investigate a potential impact of drugs on the cellular kinome. Certain methods are based on the phosphorylation of kinase substrates in the presence or absence of a drug, allowing indirect identification of alterations in kinase pathways (Dussaq et al., 2018). Other methods directly investigate the binding of compounds to recombinant kinases, typically measuring the competitive displacement of a non-selective kinase binder by the respective drug.

In this study, we have investigated the action of phenazopyridine on the cellular kinome. We demonstrate time-dependent alterations of the cellular kinome by the compound and evidence that phenazopyridine binds to several kinases, notably phosphatidylinositol kinases involved in nociception.

## Material and Methods

### Reagents

Phenazopyridine, PIK93, NIH12848, and gefitinib were purchased from Sigma. UNC3230 was obtained from RnDSystems. The anti-LC3B antibody was bought from Thermofisher, and anti-AKT and anti-phospho AKT were purchased from Cell Signaling.

### Pluripotent Stem Cells Culture and Differentiation

The CGR8 mouse PSC were cultured as described (Suter et al., 2009). For neural induction, CGR8 cells were plated in 96-well plates at a density of 10,000 cells/cm^2^ on gelatine-coated cell culture plates, in differentiation medium [BHK-21 medium supplemented with 20% foetal calf serum (Thermofisher), L-glutamine 2 mM (Thermofisher), non-essential amino acids (1X, Thermofisher), sodium pyruvate 1 mM (Thermofisher), penicillin/streptomycin 50 U/ml (Thermofisher)]. CGR8_EF1αS,RLuc-Tα1,FLuc_ cells were described in (Suter et al., 2009) and were cultured as described above. Luminescence assays were directly performed in 96-well plates with the Dual Luciferase Reporter Assay System (Promega) according to the manufacturer’s instructions. Luminescence was measured with the SpectraMax L Microplate luminometer (Molecular Devices). Cell viability in the wells was measured by the addition of propidium iodide 1 µg/ml in 100 µL of PBS and fluorescence was assessed in each well using a FluoStar Optima reader (Pharmaceutical Technology Group at the University of Geneva).

### Inverse Virtual Screening

For inverse virtual screening, the sc-PDB (Paul et al., 2004), an annotated database of druggable binding sites, was used as a target and co-crystallized ligand database. In its 2008 version, it contained more than 5,000 unique 3D binding sites from the PDB. Active sites and ligands were downloaded from http://bioinfo-pharma.ustrasbg.fr/scPDB/in mol2 format, split, and further targets were added based on the biological context or the controls required. Some proteins fitting into the biological context, but absent from the sc-PDB because of a resolution >2.5 Å, were manually added: the human tau protein kinase 1, the glycogen synthase kinase-3, the mitogen-activated protein (MAP) kinase kinase 1, the p21 activated kinase 4, MNK2, AKT2 (phenazopyridine can be superimposed to some extent with the co-crystallized ligands), and some PIM1 structures. The crystal structures of GAK, PIP4K2C, and PI4KB were not in the 2008 version of the target database because they were either not yet resolved or had a worse resolution than the cut-off mentioned. This points to a limitation of inverse virtual screening: Targets can only be found if they are included in the database. To confirm the binding modes of phenazopyridine within GAK, PIP4K2C, and PI4KB, we therefore performed comparative docking with the GOLD suite v5.2.2 (Cambridge Crystallographic Data Centre) and Glide included in the Maestro release 2020–1 (Schrödinger, Inc.). The proteins with the PDB identifiers 4Y8D (GAK), 6GL3 (PI4KB), and 2GK9 (PIP4K2C), were imported into GOLD, respectively. Hydrogens were added and water molecules were deleted. For 4Y8D and 6GL3, the respective binding site was defined by all atoms within 6.5 Å of the co-crystallized ligand. For the apo structure 2GK9, the binding site was defined by all atoms within 10 Å from Met206. Flipping of planar trigonal nitrogen atoms between cis and trans was allowed in the ligands (i.e. phenazopyridine and the co-crystallized ligands, respectively). The GOLD fitness score was used to score the protein-ligand interactions. The root-mean square deviation (RMSD) between the co-crystallized ligand and the best-scored docked ligand was calculated with the smart_rms utility of GOLD. Glide docking was performed using the same PDB structures, which were imported into Maestro and prepared with the Protein Preparation Wizard. Default settings were used to add hydrogens, assign bond orders, create disulfide bonds, and fill in missing side chains as well as loops with Prime. H-bonds were optimized using PROPKA at a default pH of 7.0. Each system was then minimized with the OPLS3e force field to an RMSD of 0.3 Å. Phenazopyridine was prepared with Ligprep using the same force field. The grids for docking were generated using the default Van der Waals radius scaling (scaling factor of 1.0 and partial charge cut-off of 0.25) and centered on the co-crystallized ligand for 4Y8D and 6GL3. For the apo structure 2GK9, the grid was centered on Asn205 and Asp218 after inferring the binding site from the holo structure of the PIP5K gamma protein, which also belongs to the PIPK family (PDB identifier: 6CMW). Ligand docking was performed with Glide XP (extra precision), allowing the ligand to be flexible. State penalties were calculated by Epik and added to the docking score. Post-docking minimization was performed with a threshold of 0.5 kcal/mol. Poses with the lowest Glide score (Gscore) were chosen as *in silico* representation of the possible interactions between phenazopyridine and each target protein.

### Kinome Scan

The kinome scan analysis was performed using the DiscoverX platform (www.discoverx.com). Briefly, T7 kinase-tagged phage strains are grown in parallel in 24-well or 96-well blocks in a BL21-derived *E. coli* host for 90 min until lysis. Lysates are centrifuged at 6,000 x g and filtered with a 0.2 µm filter to remove cell debris. Streptavidin-coated magnetic beads are treated with biotinylated kinase ligands for 30 min at room temperature to generate the affinity resin. Bound beads are blocked with excess biotin and washed with blocking buffer (SeaBlock (Pierce), 1% BSA, 0.05% Tween 20, and 1 mM DTT) to remove unbound ligand and reduce nonspecific phage binding. Phage lysates, bound affinity beads, and test compounds are combined into binding buffer (20% SeaBlock, 0.17 × PBS, 0.05% Tween 20, and 6 mM DTT) in 96-well plates. The final concentration of test compound is 10 µM. Assay plates are incubated at room temperature with shaking for 1 h. Affinity beads are treated four times with washing buffer (1X PBS, 0.05% Tween 20, and 1 mM DTT) to remove unbound phage. Beads are resuspended after a final wash in elution buffer (1X PBS, 0.05% Tween 20, and 2 mM nonbiotinylated affinity ligand) and incubated for 30 min at room temperature. Q RT PCR is used to measure the amount of phage in each eluate (which is proportional to the amount of kinase bound). Data is presented as the percentage of kinase bound by 10 µM of the ligand compared to the DMSO only as a control.

### Protein Kinase Assay

The assay was performed by using the Protein Tyrosine Kinase Assay With Cell Lysates (Pam Gene) and the Protein Serine/Threonine Kinase Assay With Cell Lysates (Pam Gene), combined together with the PamStation®12 (Pam Gene). The assay was carried out according to the manufacturer’s instructions. Data were analysed via an *in-house* software (READS Unit, Faculty of Medicine, University of Geneva): After measuring the fluorescent signal at three different time points, the slope was defined. The cut-off for being a target was defined by slopes >0.4.

### Immunostaining

Cells were fixed with PBS containing 0.5% of para-formaldehyde for 15 min at room temperature. After three washing steps in PBS, cells were permeabilized with PBS containing 0.3% of Triton X-100 (Sigma) for 1 h at room temperature. After washing with PBS three times, cells were incubated overnight with the anti-LC3B antibody in PBS with 1% bovine serum albumin (Sigma) at 4°C. After three washing steps in PBS, cells were incubated with the fluorescent secondary antibody for 90 min at 4°C in PBS with 1% bovine serum albumin. They were then incubated with 300 nm DAPI (Thermofisher) in PBS for 15 min. After washing with PBS, cells were rinsed once in water prior to mounting in the FluorSave reagent (Millipore).

### ATP Quantification

Cell viability was measured in PSC by using the ATPlite Luminescence Assay System (Perkin Elmer). PSC were plated in gelatine-coated 96-well plates at a density of 10,000 cells-cm^2^ in their standard medium. At different time points, the substrate for emission of luminescence in the presence of ATP was added according to the manufacturer’s instructions. Luminescence was measured with the SpectraMax L Microplate Luminometer (Molecular Devices).

## Results

### Data Mining for Molecular/Cellular Targets of Phenazopyridine

**FIGURE 1:**
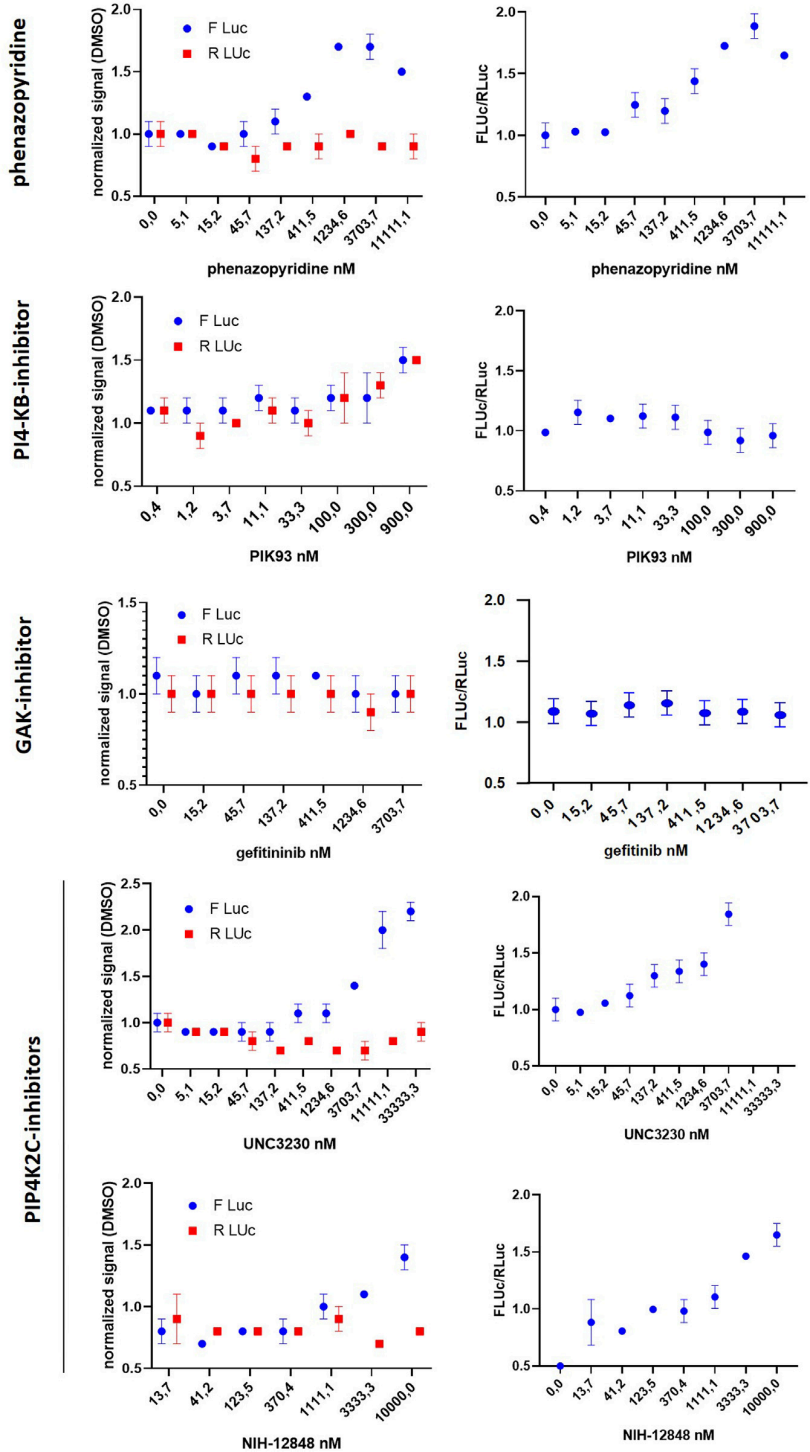
phenazopyridine regulates the human kinome. **(A)** Chemical structure of phenazopyridine. **(B)** The impact of phenazopyridine on kinases and their respective pathways was analysed in PSC by the Tyrosine or Serine/Threonine kinase assays (Pamgene). Substrates of336 kinases were exposed to the cellular content of PSC exposed to the drug. Phosphorylation of these substrates, as an indication of kinase activity, was measured by fluorescent antibodies at different time points. The fold change between phenazopyridine and DMSO control conditions was calculated. Kinases altered by phenazopyridine with a significant regulation of fold change (*p* < 0,05) were then hierarchically clustered. **(C)** Functional clustering into networks of kinases upregulated in the presence of phenazopyridine, according to an analysis with the STRING database. **(D)** Functional clustering into networks of kinases down-regulated in the presence of phenazopyridine.

A previous study from our group reported cellular effects of phenazopyridine on PSC, without an analysis of its molecular targets (Suter et al., 2009). At first, a search was performed to identify unpublished studies or screening assays reporting targets of the drug. A combined screen was then done by using the PubChem data base (www.pubchem.ncbi.nlm.nih.gov) and the ChEMBL database of bioactive molecules with drug-like properties (www.ebi.ac.uk/chembl/). ChEMBL reported 103 assays with eight of them suggesting activity of phenazopyridine, and PubChem eight activity reports among 426 bioassays (Table 1). The concentration range of the drug was between 0.1 and 10 µM. Among the reported targets, no kinases were found, but several intracellular pathways suggested their implication. The hypothesis on the interaction of phenazopyridine with kinases was reinforced by its chemical structure (Figure 1A): a structure-activity relationship analysis evidenced that the 2,6-diamino-pyridine ring is essential for activity (data will be published elsewhere in due time).

**TABLE 1:** Data mining (PubChem and ChEMBL): Reported activity of phenazopyridine on targets.

ChEMBL bioassay Id	Target or Pathway	Species	Concentration
CHEMBL1613769	Cruzain	*Trypanosoma cruzi*	20 µM
CHEMBL1794542	Estrogen receptor alpha signaling	*Homo sapiens*	3.1 µM
CHEMBL1614421	Tau Fibril Formation, Thioflavin T Binding	*Homo sapiens*	3.9 µM
CHEMBL1614240	Mitochondrial membrane potential	*Homo sapiens*	16.7 µM
CHEMBL1614204	Hemoglobin beta chain splicing at IVS2 705 locus	*Homo sapiens*	20 µM
CHEMBL2114849	Aryl hydrocarbon receptor (AhR) signaling pathway	*Homo sapiens*	39.8 µM
CHEMBL2114797	Rat pregnane X receptor (rPXR) signaling pathway	*Rattus norvegicus*	7 µM
CHEMBL3215112	Pregnane X receptor (PXR) signaling pathway	*Homo sapiens*	7.9 µM
**PubChem Bioassay ID**	**Target name**	**Species**	**Concentration**
410	Cytochrome P450 1A2	*Homo sapiens*	Na
596	Microtubule-associated protein tau	*Homo sapiens*	Na
884	Cytochrome P450 3A4 isoform 1	*Homo sapiens*	0.1 µM
915	Hypoxia-inducible factor 1, alpha subunit	*Homo sapiens*	0.5 µM
1,851	Cytochrome P450 1A2	*Homo sapiens*	Na
504,332	Euchromatic histone-lysine N-methyltransferase 2	*Homo sapiens*	10 µM
1,259,343	Mycobacterium tuberculosis	*Mycobacterium tuberculosis*	Na
504,749	Plasmodium falciparum proliferation	*Trypanosoma cruzi*	Na

To analyse whether cellular pathways are modified by phenazopyridine, a functional protein kinase analysis (Pamgene, www.pamgene.com) was performed on PSC exposed to 10 µM of phenazopyridine. The tyrosine kinase array consists of 196 peptides with known phosphorylation sites, representing 100 different proteins correlated with one or multiple upstream kinases. The serine/threonine kinase array consists of 140 Ser/Thr containing peptides, together representing 60% of the human kinome. Supplementary Table S1 shows the used peptides and associated Uniprot numbers. The PSC content was exposed to these peptide libraries and a phosphorylation analysis (by antibodies against phosphoproteins) was performed at four different time points (10 min, 1 h, 3 h, and 3 d). Variations in target phosphorylation between phenazopyridine and DMSO were calculated by a fold change analysis: 68 out of 336 targets showed variations in fold change between DMSO and phenazopyridine (Figure 1B). Raw data are shown in the Supplementary Table S2 and, because one peptide can be targeted by several pathways, the correspondences between the Uniprot number of the peptides and the targets is shown in Supplementary Table S3. Regulations by phenazopyridine started early on (after 10 min) and evolved dynamically until three days of exposure. Among the targets regulated by phenazopyridine, we detected different kinase groups (Figures 1C,D) and grouped them functionally into networks via the functional protein association network STRING (www.string-db.org). After 10 min, the proto-oncogenes YES1 and ABL-1, which are involved in the cell cycle, were upregulated (Figure 1C), whereas several kinases were rapidly down-regulated (after 10 min), with several kinases belonging to the AKT pathway (Figure 1D). The AKT pathway promotes cell survival, growth, and migration (Lamalice et al., 2007). *It also* inhibits autophagy (Palmieri et al., 2017). Although Phosphatidyl-Inositol Kinases (PIK) are the major AKT activators, other kinases, including calcium/calmodulin-dependent Kinases (CAMK), activate AKT directly (Soderling, 1999; Vanhaesebroeck and Alessi, 2000; Mahajan and Mahajan, 2012). After 1 h, particularly kinase groups involved in cell proliferation and differentiation were upregulated, notably MAPK and CDK (Figure 1C). After three days, multiple regulations were linkedparticularly to a network which involved CAMK, which is also mainly involved in cell cycle and cytoskeletal rearrangement.

**FIGURE 2:**
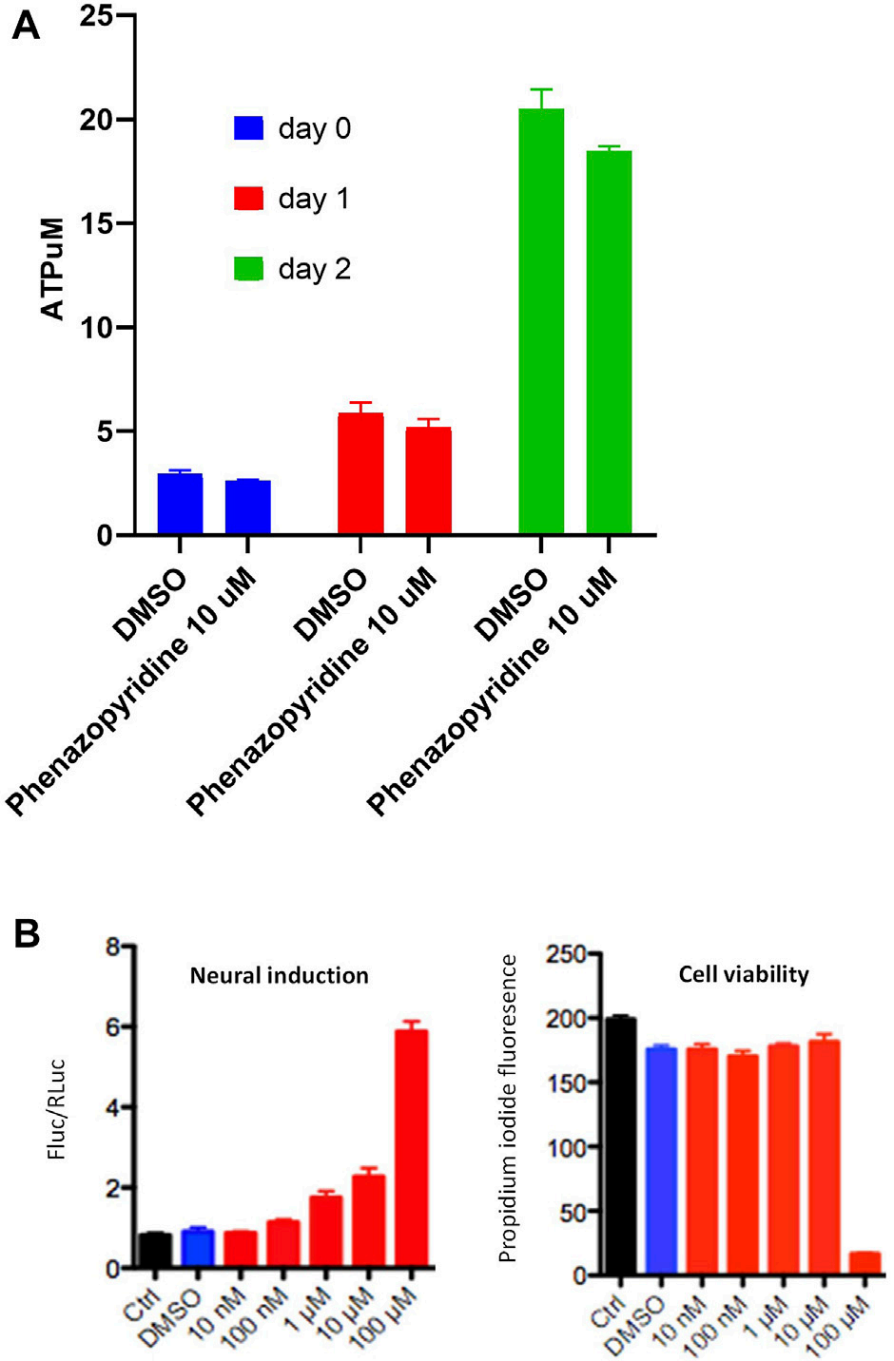
phenazopyridine induces differentiation of PSC with no effects on proliferation. **(A)** The CGR8 PSC line was exposed to phenazopyridine at 10 µM or DMSO control conditions. After two days, cells were lysed for the quantification of intracellular ATP. **(B)** CGR8_EF1αS,RLuc-Tα1,FLuc_ cells were exposed to phenazopyridine at various concentrations or DMSO control conditions. After three days, cells were lysed for the quantification of F-luminescence (FLuc) and R-luminescence (RLuc) in the presence of the respective substrates. The left panel shows the ratio of luminescence intensity between FLuc and RLuc for different concentrations of phenazopyridine. The right panel shows cell viability for various phenazopyridine concentrations, assessed by analysis of the fluorescence in the presence of propidium iodide.

All these observations made in PSC show a functional interference of phenazopyridine with several groups of kinases mainly involved in cell proliferation/differentiation processes, with notably a reduction of the AKT pathway. Accordingly, PSC exposed to 10 µM of phenazopyridine confirmed at the protein level a reduced phosphorylation of AKT at 10 min, 1 and 3 h after exposure (Supplementary Figure S1). It is noteworthy that, in parallel, a regulation of the ERK pathway was not confirmed at the protein level, assessed by the study of ERK phosphorylation by Western blotting (data not shown). The effects of the drug on proliferation or differentiation was next investigated in quantitative assays. The rate of cell proliferation of the PSC, assessed during five days by quantification of intracellular ATP, was not affected by the drug (Figure 2A). The CGR8 PSC line was stably transduced with a dual promoter/reporter gene construct containing a ubiquitous promoter (EF1αS) controlling the expression of Renilla Luciferase (RLuc) and also an early neural-specific promoter (Tα1), controlling the expression of Firefly Luciferase (FLuc). Then, neural differentiation was quantified by the normalized ratio between FLuc and RLuc (Suter et al., 2009). CGR8_EF1αS,RLuc-Tα1,FLuc_ cells were exposed to phenazopyridine at different concentrations. After 72 h, the cells were lysed for RLuc and FLuc luminescence measurement in the presence of their substrates, each signal being normalized with the signal obtained with the vehicle alone (DMSO). An increase of the FLuc signal was observed at 1 µM of the phenazopyridine, with a maximal signal at 10 µM (Figure 2B) without any toxicity (i.e. no signal in the presence of propidium iodide).

Our observations made in PSC evidenced thus a functional interference of phenazopyridine with several groups of kinases mainly involved in cell proliferation/differentiation processes, with notably a reduction of the AKT activation. Accordingly, PSC exposed to 10 µM of the drug did not proliferate but underwent differentiation.

### *In silico* Inverse Screening for Molecular Targets of Phenazopyridine Suggests Binding of the Drug to Kinases

*In silico* inverse screening is a ligand-driven methodology aiming at identifying a specific target for a given ligand. Here, target prioritization happened on a multi-criteria basis and a target was selected if four of the following criteria were fulfilled, with criterion 3) being mandatory: 1) 50% of the druggable binding site database (sc-PDB) entries for a particular target were scored among the top 2% scoring entries, 2) the average GOLD fitness score, indicating how well phenazopyridine interacts with a target, was above 50 for all entries of the corresponding target, 3) the keyword search in the PDB and the literature search revealed a link with neurogenesis, and 4) the docking accuracy, i.e. the RMSD to the co-crystallized ligand was preferably below 2 Å. Ranking of the targets according to the docking score yielded a list of putative kinase targets, with a high score indicating a better predicted protein-ligand binding energy, and therefore a higher likelihood for phenazopyridine to bind strongly to a given target. Table 2 contains for each kinase the name and acronym, the PDB identifier of the best-ranked structure, the ranking position in percent, the heavy atom RMSD of the co-crystallized ligand as a control, and the experimental confirmation from the kinome scan by Ambit Biosciences (http://www.ambitbio.com/) performed at the University of Dundee according to a previously described procedure (Bain et al., 2003). For Abl, the IC_50_ value of phenazopyridine was determined at the University of Milano-Bicocca as detailed in Gunby et al. (Gunby et al., 2005). The target prediction success rate, i.e. the number of predicted vs. the number of experimentally confirmed targets is 80% when only considering the targets in the top 10% of the phenazopyridine score-based ranking and 71–83% when considering all true positives of the seven kinases in Table 2 (not including or including Abl).

**TABLE 2:** *In silico* inverse screening. Names and acronyms of kinase targets included in the inverse screening and experimentally tested. “Rank (%)” means that a potential target is found in the top N% of the phenazopyridine score-based ranking. wt stands for wild type protein and n/d for not determined*.*

Name (acronym)	PDB id	Rank (%)	Heavy atom RMSD \of co-crystallized ligand (Å)	Kinome scan (% of control at 10 µM)	Dundee activity test (% of remaining activity at 10 µM)	Milano activity test	Exp. confirmed target
Proto-oncogen Ser/Thr kinase PIM1 (PIM1)	2O65 and others	0.8	3.5	100	49	n/d	+
Checkpoint kinase 1 (CHK1)	2CGU and others	3.6	2.8	100	Nd	n/d	−
Abelson (Abl)	1FPU and others	4.5	1.6	48	Nd	Phenazopyridine is inactive at 100 µM	(+)
Stem cell factor receptor (c-KIT)	1PKG	6.0	5.4	41 (wt), 32 (Val559Asp, best mutant)	Nd	n/d	+
Phosphatidylinositol-4,5-bisphosphate 3-kinase γ (PIK3CG)	2A5U and others	6.1	2.3	27	Nd	n/d	+
Mitogen-activating protein kinase interacting Ser/Thr kinase (MNK2)	2HW7	30.3	0.6	6.6	20	n/d	+
Casein kinase 1 isoform γ 3 (CK1)	2IZU	74.6	0.4	44	Nd	n/d	+

### Phenazopyridine Binds to Recombinant Human Kinases

The physical interactions of phenazopyridine with cellular kinases was next investigated by an active site-directed competition binding assay for quantitatively measuring the interactions between phenazopyridine and 401 individual human kinases. If phenazopyridine interacts physically with a defined kinase (directly or allosterically), it prevents the binding of beads coated with staurosporine, a non-specific inhibitor of kinases. Hits are identified by an ultra-sensitive quantitative PCR detecting the specific DNA tag linked to each kinase (Supplementary Figure S2). The drug is used at a concentration of 10 µM and results are expressed as the percentage of signal (kinase PCR detection) with respect to DMSO control conditions. Thirteen hits were identified, with a signal reduction ranging from 0 to 27% (Table 3). Full raw data are presented in Supplementary Table S4. Interactions between 0 and 20% of reduction were considered to be the most significant ones. Then, eight interactions of phenazopyridine with kinases were considered: two with PIK proteins (PIP4K2C, PI4KB), and one with MAPK (MKNK2), RIOK2, TYK2, ANKK1, DYRK2, and the cyclin-associated GAK, respectively. RIOK2 showed the best score with a complete inhibition of kinase binding to staurosporine. To calculate the binding affinity of phenazopyridine for the kinases corresponding to the eight hits, quantitative experiments, namely a *K*_*d*_ *select* analysis, were performed via the DiscoverX technology by testing different concentrations of the compound and calculating the equilibrium dissociation constant K_d_. A sub-micromolar affinity (K_d_<1 µM) was only seen with two PIKs (PIP4K2C, PI4KB) and GAK (Table 4). Inhibitions curves from these compounds are shown in Supplementary Figure S3.

**TABLE 3:** Binding of phenazopyridine to human kinases. Phenazopyridine was used at 10 µM. The negative control was DMSO, the positive control a compound provided by DiscoverX, inducing 100% of release of the kinase from the beads. Results are expressed as the percentage of signal corresponding to the negative control, according the following formula: [(signal compound—signal positive control)/(signal negative control—signal positive control)] x 100.

Target	% Of control
RIOK2	0
PIP5K2C	2,3
PIK4CB	4,6
MKNK2	6,6
GAK	16
TYK2	16
ANKK1	18
DYRK2	20
FLT3	21
PIK3CB	22
PIK3C2G	24
FLT3	25
PIK3CG	27

**TABLE 4:** Determination of the K_d_ for the interaction between phenazopyridine and eight selected kinases.

Kinase name	Phenazopyridine K_d_ (nM)
RIOK2	1,000
PIP4K2C	540
PI4KB	730
MKNK2	2,100
GAK	760
TYK2	26,000
ANKK1	21,000
DYRK2	5,000

### *In silico* Confirmation of Phenazopyridine Binding to the Three Selected Kinases

**FIGURE 3:**
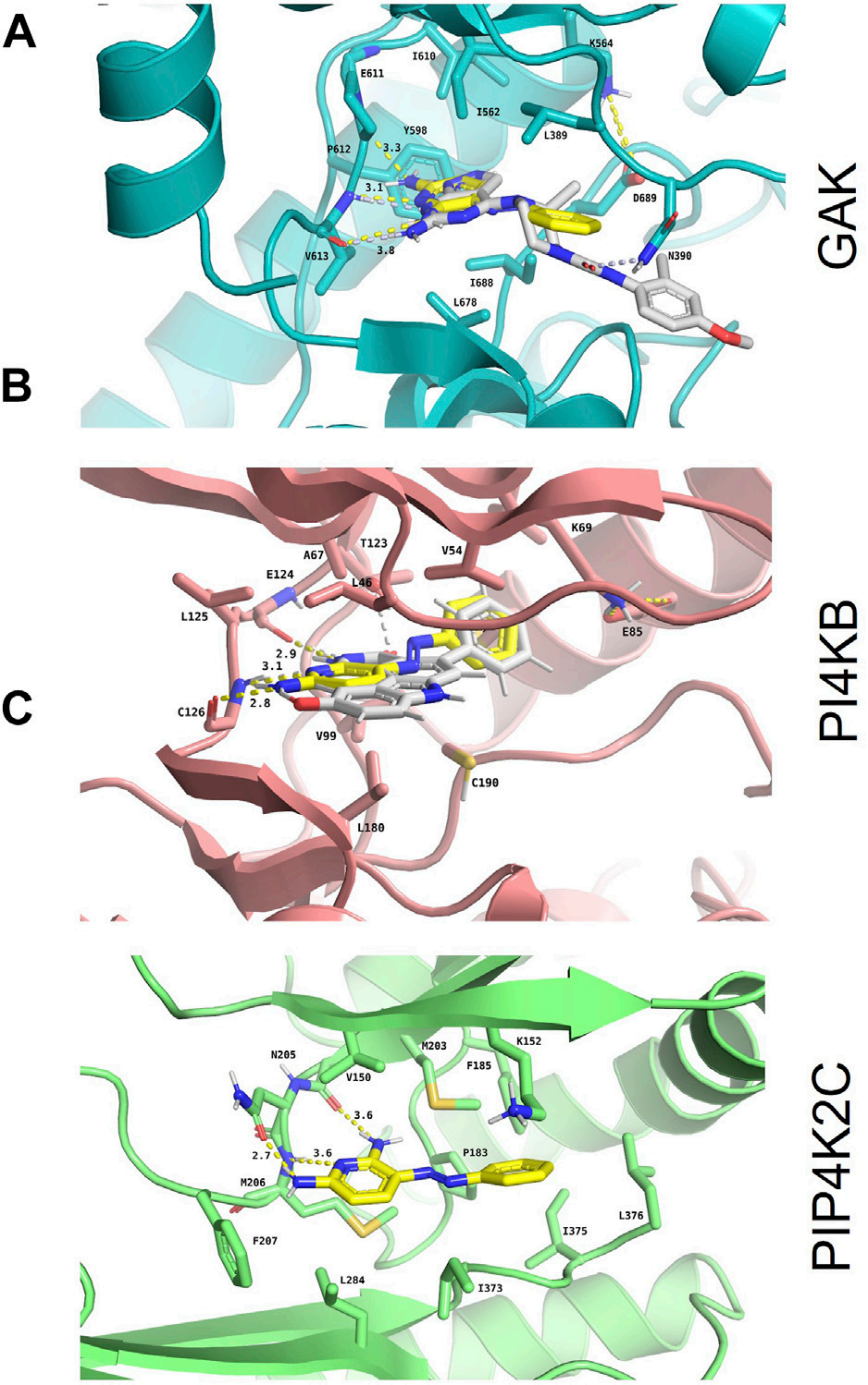
*In silico* confirmation of phenazopyridine binding to the three selected kinases. Binding mode and interaction diagrams of phenazopyridine within **(A)** human cyclin-G-associated kinase (GAK; PDB identifier: 4Y8D), **(B)** phosphatidylinositol 4-kinase *β* (PI4KB; PDB identifier: 6GL3), and **(C)** phosphatidylinositol-5-phosphate four kinase type 2 gamma (PIP4K2C; PDB identifier: 2GK9). The atoms of phenazopyridine are shown as sticks with yellow carbons and the interacting residues of each protein as sticks with orange **(A)**, blue **(B)**, and brown carbons **(C)**, respectively. For the holo crystal structures **(A)** and **(B)**, the carbon atoms of the respective co-crystallized ligands are displayed with white sticks. H-bonds are shown as dashed yellow lines, and their lengths are indicated in Ångströms. Binding site residues directly interacting with phenazopyridine and the catalytic Lys are labelled as well.

The binding modes of phenazopyridine with the best predicted interaction energies (best-scored poses) within the ATP-binding pockets of each of the three kinases were obtained by docking. All docked poses of phenazopyridine obtained with GOLD and GLIDE were compared visually and the heavy atom RMSD among all solutions was below 1.0 Å, indicating highly similar docking poses. In the three kinases, phenazopyridine displays a strong anchoring to the so-called hinge region via two to three H-bonds (Figures 3A–C). The molecule is further stabilized by hydrophobic interactions contributed by the top (P-loop) and the bottom of each binding site. Besides, the area occupied by phenazopyridine in each kinase overlaps well with each co-crystallized ligand, which means that each crystallized kinase conformation can accommodate phenazopyridine well, even if the co-crystallized ligands of GAK and PI4KB received slightly higher GOLD fitness scores than phenazopyridine (56.61 and 63.86 for the co-crystallized ligands, respectively, vs. 46.20 and 43.23 for phenazopyridine). Compared to the overall score-based ranking of the inverse screening, the interaction scores of phenazopyridine for the three kinases would rank in the first 25.9% (PIP4K2C), 53.5% (PI4KB), and 67.8% (GAK) of the putative target list.

### Phenazopyridine Induces an Autophagic Response Without Cell Death, Similarly to PIP4K2C Inhibitors

**FIGURE 4:**
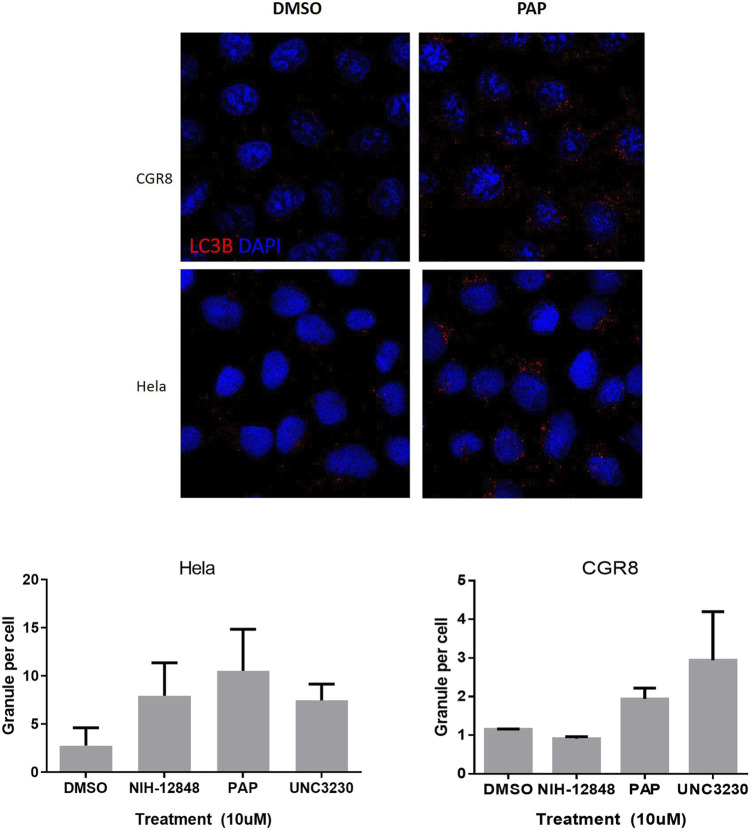
Analysis of PSC autophagy in the presence of phenazopyridine. PSC or HeLa cells were exposed for three days to 10 µM of phenazopyridine or PIP4K2C inhibitors, prior to analysis and quantification of LC3B positive autophagosomes by immunofluorescent staining.

Autophagy is a process characterized by the appearance of vacuoles (autophagosomes), leading to self-digestion of cellular components, notably in response to stress (Degtyarev et al., 2008) or xenobiotics/drugs (Bolt and Klimecki, 2012). Depending of these transitory environmental conditions, autophagy can either promote survival or apoptosis (Degtyarev et al., 2008). PIK/AKT pathway inhibition has been reported to increase cell autophagy (Palmieri et al., 2017). More specifically, PIP4K2C has been described as a down-regulator of autophagy (Sharma et al., 2019). Based on the observation that phenazopyridine reduces AKT signalling via two PIKs, namely PIP4K2C and PIK4KB, autophagosomes were measured after exposure to the drug. In a similar way as for UNC-3230, an inhibitor of PIP4K2C (Wright et al., 2014), the number of LC3B-positive autophagosomes was increased three days after phenazopyridine exposure, in PSC and HeLa cells (Figure 4). This observation was confirmed in HeLa cells, but not in PSC, using the PIP4K2C inhibitor NIH-12848 (Clarke et al., 2015) (Figure 4). Drug-induced autophagy did not result in cell death, because PSC exposed to the drug for three days showed unchanged ATP levels and therefore unaltered cell growth.

## Discussion

The mechanism of action of phenazopyridine is unknown. Kinases were not detected as targets by data mining, probably because they have not been investigated previously as potential targets. The Pamgene analysis provided the first evidence that the drug is regulating the kinome, whereas *in silico* screening and kinome scans detected physical interactions between the drug and individual targets.

In the *in-silico* analysis, two kinases were detected by the Dundee screening: PIM1 and Abl. For Abl, we observed a discrepancy between the kinome scan data and the activity data: While the kinome scan indicated that Abl with phenazopyridine has 48% of remaining activity when compared to the control at 10 μM, the activity assay data indicate that wild-type Abl is not inhibited by phenazopyridine up to 100 µM and causes 35% inhibition at 500 µM (data not shown). This discrepancy can potentially be explained in the following way: Phenazopyridine binds to Abl but does not inhibit it (at least at concentrations below 500 µM), similarly to the allosteric Abl inhibitor GNF-2 (a pyrimidine benzamide).

In addition, some discrepancies were noticed in the comparison between *in silico* screening and the DiscoveryX kinome scan. Some targets (CHK1 and PIM1) are present in the *in-silico* screening but not in the DiscoveryX kinome scan, which detects competition between the drug and the native ligand staurosporine. We hypothesize that phenazopyridine interacts probably with the kinase at the same site than staurosporine, but does not compete enough with it to displace it. On the contrary, some targets were found in the kinome scan but not *in silico*: These kinase binding sites were either not part of the database used for inverse virtual screening, the corresponding X-ray structures had a low resolution, or no X-ray structure had been solved.

Particularly the PIK/AKT pathway was targeted by phenazopyridine. This pathway is important for many cellular functions such as cell growth, metabolism, and most importantly, nociception (Wright et al., 2014). Notably, numerous pain-producing receptors signal via the hydrolysis of phospholipids (Wright et al., 2014). Two PIKs are inhibited by phenazopyridine with a sub-micromolar affinity: PI4KB and PIP4K2C. PI4KB regulates the trafficking from the Golgi system to the plasma membrane. Nevertheless, its nuclear function is unknown. PIP4K2C is poorly characterized. It is a PIK from a family that contains three members: It phosphorylates the phosphatidyl-inositol (PtdIns)5P, producing PtdIns4,5P_2_ (or PIP2), which is important for PIK signal transduction pathways (McCrea and De Camilli, 2009; Fiume et al., 2015). The functions of PIP4K2C are poorly known, although mutations suggest that this kinase could be involved in cell proliferation and acute myeloblastic leukaemia (Lima et al., 2019). However, it has been linked to the autophagic response (Vicinanza, 2015 #64). In addition to providing a new inhibitor of PIP4K2C, we identified a new link between the inhibition of this enzyme and early neural development of ESC. By reducing AKT phosphorylation in ESC exposed to phenazopyridine (because activation of PIK pathways results in AKT phosphorylation), the PIP4K2C/AKT pathway is suggested to be involved in this process.

Interestingly, among all the kinases inhibited by phenazopyridine, PIP4K2C inhibition has been shown to harbour analgesic effects. Indeed, regarding nociceptive sensitization, it is known that drugs that block signalling linked to MAPK reduce pain in animal models (Aley et al., 2000; Aley et al., 2001; Dai et al., 2002; Ji et al., 2002; Cheng and Ji, 2008; Ji et al., 2009). Moreover, the hydrolysis of the phospholipid PIP2 produces products regulating nociceptive sensitization (Gamper and Shapiro, 2007; Rohacs et al., 2008; Falkenburger et al., 2010; Gold and Gebhart, 2010; Tappe-Theodor et al., 2012). Interestingly, inhibiting the kinases that generate PIP2 in neurons was shown to reduce pain, notably PIP5K1C and PIP4K2C. It has been notably validated that inhibition of PIP5K1C is analgesic and attenuates pain in animal models. Also, UNC-3230, the inhibitor of PIP4K2C, lowers PIP_2_ in neurons and attenuates pain (Wright et al., 2014). Thus, inhibition of PIP4K2C was directly linked to analgesic effect, making a solid link between the mechanism of action of phenazopyridine and its therapeutic use (Wright et al., 2014).

GAK is a serine/threonine kinase involved in vesicle transport. It is inhibited by phenazopyridine and has emerged as a promising target for the treatment of viral infections. However, few potent and selective GAK inhibitors have been reported. Isothiazolo (5,4-*b*) pyridines were discovered as selective GAK inhibitors with potent activities against the hepatitis C virus (Kovackova et al., 2015). These GAK inhibitors also represent tools to study GAK function in different disease areas where GAK is implicated (i.e. viral infections, cancer, and Parkinson’s disease). Phenazopyridine showed a sub-micromolar affinity inGAK (K_d_ = 760 nm).

Other kinases were inhibited by phenazopyridine with a lower affinity. RIOK2 is an atypical kinase of the RIO kinases family, which is involved in ribosome biogenesis. This kinase is necessary for the maturation of the pre-40S particle (small ribosomal subunit) in human and yeast cells (Ferreira-Cerca et al., 2012). It has also been reported to be involved in cell cycle progression. Its overexpression enhances tumorigenesis in murine astrocytes by up-regulating AKT-signaling (Read et al., 2013). Accordingly, RIOKs are involved in a variety of human cancers including colorectal carcinoma, melanoma, non-small-cell lung carcinoma, and glioblastoma (Berto et al., 2019). Despite these reported roles, the RIOK pathway remains poorly understood. Therefore, chemical tools targeting RIOKs are still needed to further elucidate their function and evaluate their potential as anticancer targets. Three compounds have been initially reported with a K_d_ less than 200 nm (Varin et al., 2015). However, they were discovered using large kinase assay panels and were not selective. Diphenpyramide and analogs were described as RIOK2 inhibitors with more selectivity, with the former having a K_d_ of 160 nm (Varin et al., 2015). Phenazopyridine is presented here as a new compound that inhibits RIOK2 with a K_d_ of 1 µM.

MKNK2 encodes a member of the CAMK Ser/Thr protein kinase family. It is one of the downstream kinases activated by MAPK. It phosphorylates the eukaryotic initiation factor 4E (eIF4E), thus playing important roles in the initiation of mRNA translation and malignant cell proliferation (Hou et al., 2012). Numerous inhibitors of MKNK2 are described.

We report an autophagy response induced by phenazopyridine, a mechanism removing unnecessary or dysfunctional components in autophagosomes (Klionsky, 2008; Mizushima and Komatsu, 2011). In addition to autophagy, neural differentiation of stem cells is favored by phenazopyridine (Suter et al., 2009). Autophagy is induced by cellular stress and exposure to drugs and xenobiotics (Degtyarev et al., 2008; Mizushima and Komatsu, 2011; Bolt and Klimecki, 2012) and often participates in normal tissue remodeling through elimination of pre-existing altered materials and creation of new components. During early development, massive autophagy plays a major role in organogenesis (Tsukamoto et al., 2008). Accordingly, autophagy is frequently observed in stem cells, including neural stem cells (Hu et al., 2019). Autophagy favors neural stem cell differentiation by degrading Notch1 into autophagosomes (Wu et al., 2016). Depending on the stimuli, autophagy can either promote survival or apoptosis (Degtyarev et al., 2008): Apoptosis is not observed after phenazopyridine exposure, and the drug is not toxic. In accordance with our observations, the PIK/AKT pathway inhibition has been reported to increase autophagy (Palmieri et al., 2017; Sharma et al., 2019). It is noteworthy that inducers of autophagy are receiving attention for addressing neurodegenerative diseases via removal of abnormal/toxic protein aggregates (Heras-Sandoval et al., 2014).

In conclusion, the action of phenazopyridine on the human kinome is described for the first time. It is a new kinase inhibitor with a sub-micromolar affinity, notably affecting kinases of the PIK family involved in nociception. The pharmacological description of phenazopyridine’s action is useful for the understanding of its mechanism of action and provides a new tool for the delineation of the biological roles of kinases.

## Data Availability

The raw data supporting the conclusions of this article will be made available by the authors, without undue reservation, to any qualified researcher.
