# Supplementary figures and images for: Novel Mechanism for an Old Drug: Phenazopyridine is a Kinase Inhibitor Affecting Autophagy and Cellular Differentiation
Source: Front Pharmacol. 2021 Aug 4;12:664608. doi: 10.3389/fphar.2021.664608 (PMC8371461; doi:10.3389/fphar.2021.664608)

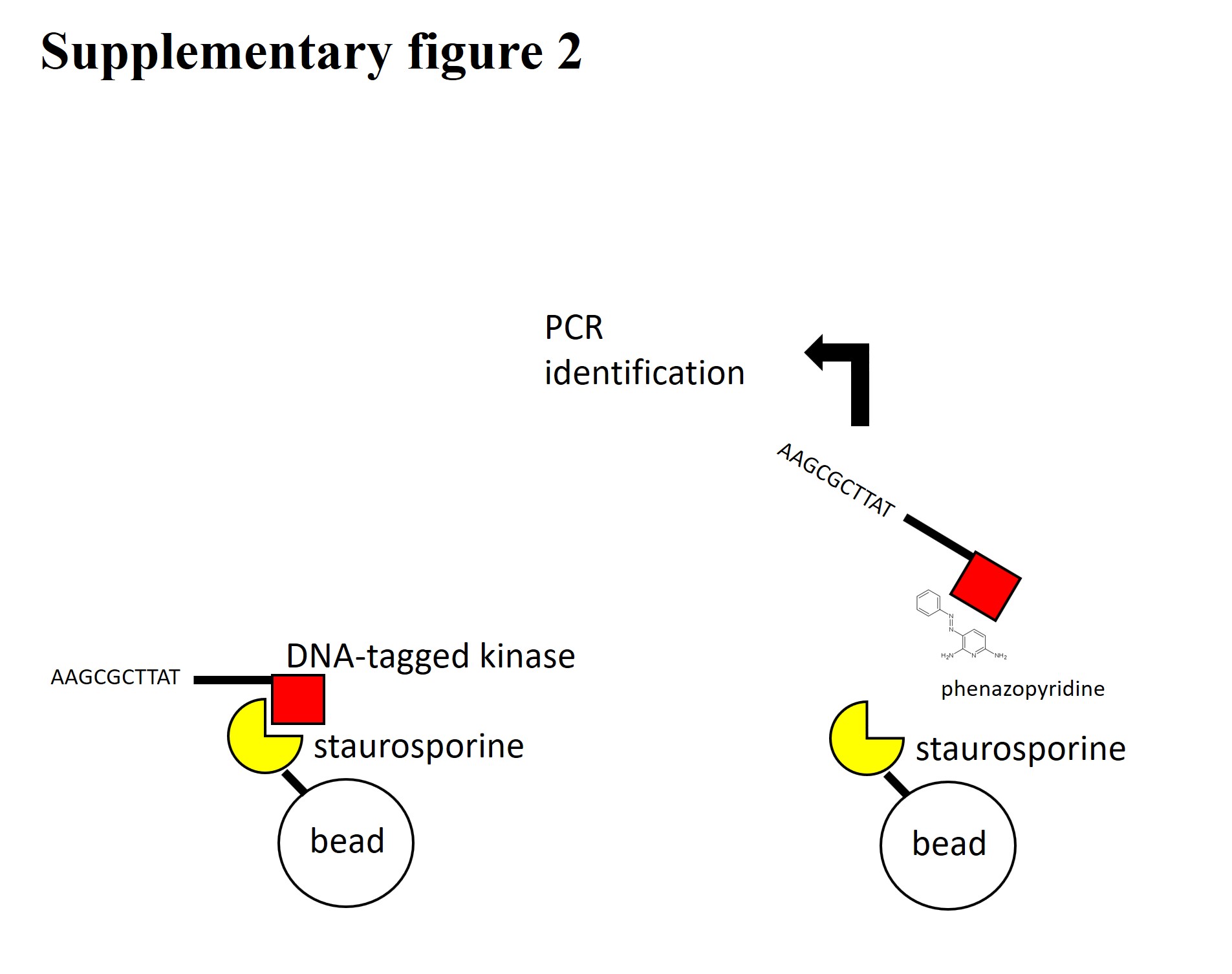

Supplement: Supplementary file 2 [file Image3.JPEG]

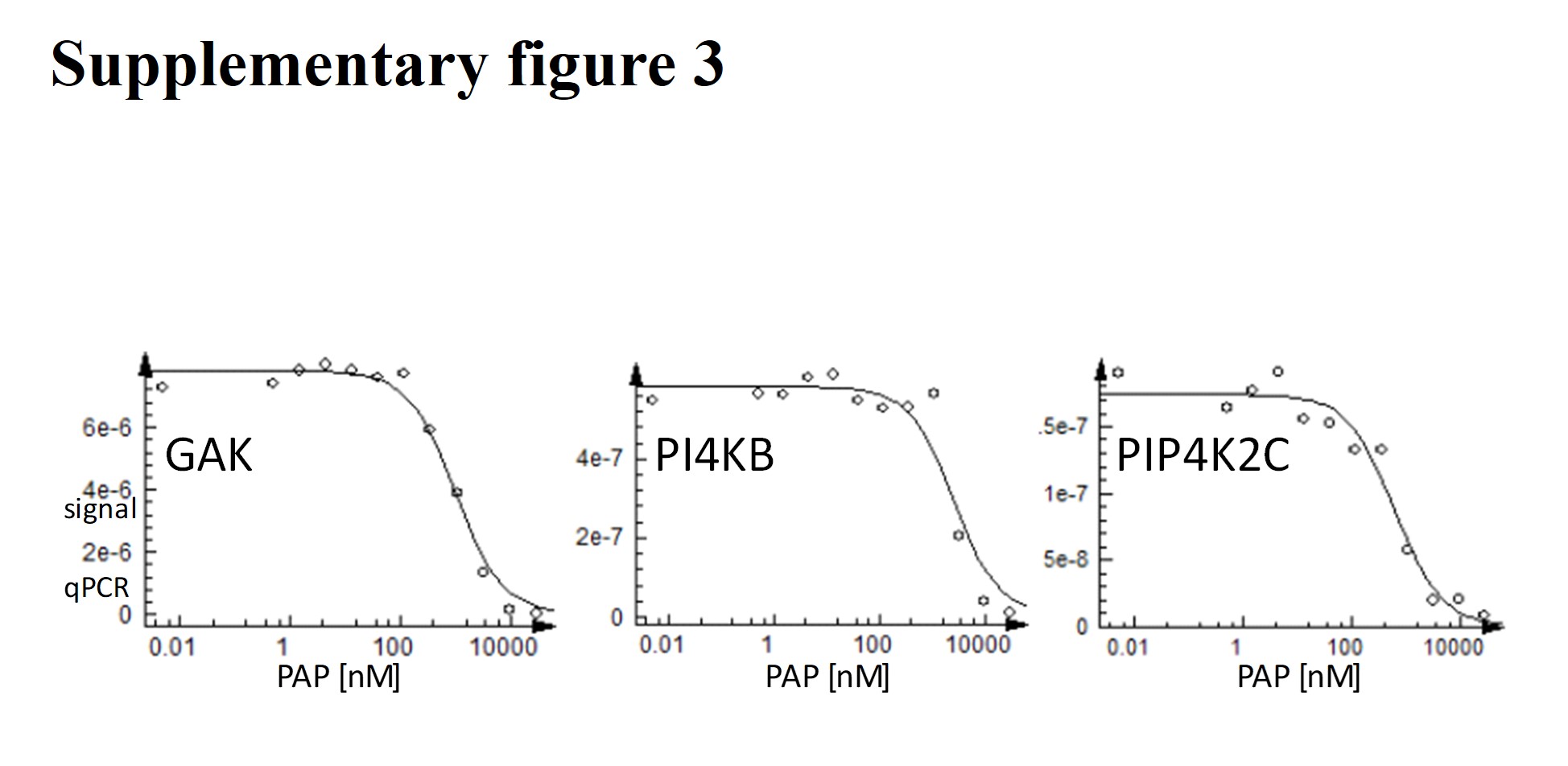

Supplement: Supplementary file 4 [file Image1.JPEG]

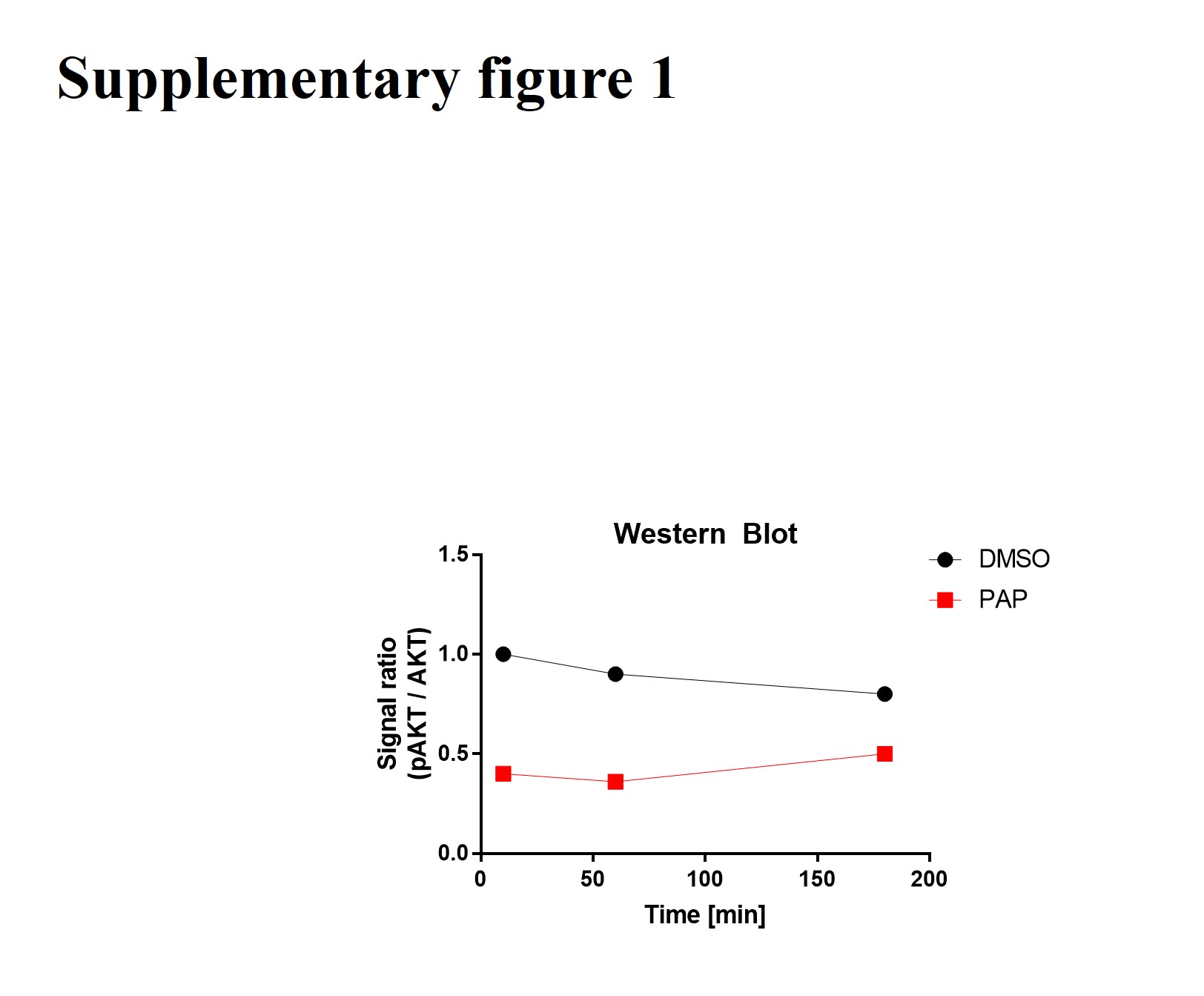

Supplement: Supplementary file 5 [file Image2.JPEG]
